# Dose to craniofacial region through portal imaging of pediatric brain tumors

**DOI:** 10.1120/jacmp.v13i1.3385

**Published:** 2012-01-05

**Authors:** Christine J. Hitchen, Etin‐Osa Osa, J. Keith Dewyngaert, Jenghwa Chang, Ashwatha Narayana

**Affiliations:** ^1^ Department of Radiation Oncology New York University Medical Center New York NY; ^2^ Department of Radiation Oncology New York‐Presbyterian Hospital/Weill Cornell Medical College New York NY USA

**Keywords:** radiation therapy, pediatric brain tumors, portal imaging, portal imaging dose, IMRT

## Abstract

The purpose of this study was to determine dose to the planning target volume (PTV) and organs at risk (OARs) from portal imaging (PI) of the craniofacial region in pediatric brain tumor patients treated with intensity‐modulated radiation therapy (IMRT). Twenty pediatric brain tumor patients were retrospectively studied. Each received portal imaging of treatment fields and orthogonal setup fields in the craniofacial region. The number of PI and monitor units used for PI were documented for each patient. Dose distributions and dose‐volume histograms were generated to quantify the maximum, minimum, and mean dose to the PTV, and the mean dose to OARs through PI acquisition. The doses resulting from PI are reported as percentage of prescribed dose. The average maximum, minimum, and mean doses to PTV from PI were 2.9±0.7%, 2.2±1.0%, and 2.5±0.7%, respectively. The mean dose to the OARs from PI were brainstem 2.8±1.1%, optic nerves/chiasm 2.6±0.9%, cochlea 2.6±0.9%, hypothalamus/pituitary 2.4±0.6%, temporal lobes 2.3±0.6%, thyroid 1.6±0.8%, and eyes 2.6±0.9%. The mean number of portal images and the mean number of PI monitor units per patient were 58.8 and 173.3, respectively. The dose from PI while treating pediatric brain tumors using IMRT is significant (2%–3% of the prescribed dose). This may result in exceeding the tolerance limit of many critical structures and lead to unwanted late complications and secondary malignancies. Dose contributions from PI should be considered in the final documented dose. Attempts must be made in PI practices to lower the imaging dose when feasible.

PACS numbers: 87.55ne, 87.55Qr

## I. INTRODUCTION

Brain tumors are the second most common cancers of childhood, after hematological malignancies, and account for approximately 20%–25% of all primary pediatric tumors.^(^ ^1^ ^)^ Although the prognosis has improved recently, brain tumors still remain the leading cause of death for all childhood cancers.^(^ ^1^ ^)^ Treatment of pediatric brain tumors usually involves a combination of surgery, radiotherapy, and chemotherapy. However, the quality of life for survivors of pediatric brain tumors is influenced by the long‐term side effects of these treatments that include neurocognitive deficiencies, endocrine dysfunction, focal neurological deficits, and psychosocial sequelae.^(^ ^2^ ^)^

Radiation therapy remains an essential part of the management of most pediatric brain tumors, even though there are new developments in neurosurgery techniques and chemotherapy armamentarium. Both the radiation dose and volume of brain that is being treated have been known to strongly correlate with long‐term side effects.^(^ ^3^ ^)^ Therefore, radiation dose to the normal tissue should be kept as low as possible to minimize side effects. In radiotherapy of pediatric brain tumors, the aim of treatment planning is to design adequate dose coverage to the planning target volume (PTV), while limiting dose to organs at risk (OARs) and normal surrounding tissue. Frequently, to achieve maximum PTV dose coverage, the treatment plan results in delivering maximum tolerance dose to OARs such as brainstem, optic nerves/chiasm, cochlea, hypothalamus/pituitary, temporal lobes, thyroid, and eyes. As a result, there has been a shift in treatment planning from conventional to three‐dimensional (3D) and, more recently, to intensity‐modulated radiation therapy (IMRT).

There is clear evidence that susceptibility to secondary cancers is much higher for those who are exposed to radiation early in life compared to those who are exposed later in life.^(^ ^4^ ^)^ The incidence of leukemia is much higher and also the type of leukemia is different among those under 20 years of age compared to those older than 20 years of age. Similarly, the risk of breast cancer and thyroid neoplasms is highest among those exposed under 20 years of age. Radiation effect has been studied over a wide range of ages and doses, and the increased incidence of carcinogenesis and mutagenesis with increasing doses has been clearly noted.^(^ ^5^ ^)^ The assumption, based on mathematical risk models, is that radiation‐induced carcinogenesis results from the activation of an oncogene, the loss of a suppressor gene, or a combination of both. While short‐term modeling includes the effects of dose, dose rate, radiation quality, and fractionation, long‐term modeling takes into consideration the age at the time of exposure and the time since the exposure. More recent studies have developed a risk model which marries short‐ and long‐term formalisms in order to predict as accurately as possible the induction of secondary malignancies from radiotherapy.^(^ ^6^ ^)^

As part of treatment verification and quality assurance during radiotherapy, the patient receives portal imaging (PI) at the beginning of the treatment, including the open‐field imaging for orthogonal setup fields, as well as double exposure imaging of each treatment field. This is followed by weekly or biweekly PI of the orthogonal setup fields to verify the treatment setup. The number of imaging fields for brain radiotherapy has increased significantly in the past few years due to the adoption of intensity‐modulated radiotherapy (IMRT) technique^(^ ^7^ ^)^ that generally uses multiple beams from various directions to better spare normal tissue. The cumulative imaging dose, while small for each exposure, might become significant in comparison to the treatment dose for fractionated radiotherapy. However, the additional dose delivered to OARs through PI is generally not included in the dose calculation of the treatment plan.

In this study, we investigate the dose to the PTV and OARs due to megavoltage (MV) PI of the craniofacial region in pediatric brain tumor patients receiving radiation therapy using IMRT. The number of portal images and monitor units used for PI were documented for each patient. A commercial RT treatment planning system was used to calculate dose distributions and dose‐volume histograms using the apertures of the PI as treatment beams. The results were analyzed and presented for different treatment locations in the brain. Means to reduce PI dose for pediatric brain patients are discussed.

## II. MATERIALS AND METHODS

Between July 2005 and April 2008, 20 pediatric patients (age range 1–20 years) received portal imaging of the craniofacial region during the course of radiation treatments at our institution. Thirteen patients received partial brain (7 brainstem and 6 nonbrainstem cases) treatments, four patients received craniospinal irradiation (CSI), and three patients received treatments to the skull base region (1 maxilla, 1 orbit, 1 nose). All treatments were planned and delivered using 4 or 10 MV beam of Varian 2100EX linear accelerator (Varian Medical System, Palo Alto, CA, USA). The prescription dose ranged from 4500 cGy to 5940 cGy, with a median prescription dose of 5580 cGy. The prescribed dose per fraction was 180 cGy. All treatment fields used intensity modulation with dynamic multileaf collimators (DMLC), with the exception that the CSI were treated with static lateral step brain fields using multileaf collimators (MLC). The CSI step brain was followed by brain boost treatment using IMRT fields with DMLC.

Megavoltage (4 MV) portal images were acquired using an aS500 electronic portal imaging device (EPID) (Varian Medical System, Palo Alto, CA, USA). Portal images were acquired of orthogonal setup fields for the first three days of treatment and weekly or biweekly thereafter. The treatment fields were imaged at the beginning of treatment. The median monitor units (MUs) per image were 3 (range 1–4). The imaging technique for the orthogonal setup fields was single exposure with the collimators opened to visualize surrounding anatomy. The treatment field imaging, on the other hand, employed a double exposure technique. The first exposure was an image of the treatment field. The second exposure was acquired with the collimators opened to visualize surrounding anatomy.

Dose distributions and dose‐volume histograms (DVHs) were generated for PTV and organs at risk (OARs) of each patient using the Eclipse treatment planning system (Varian Medical System, Palo Alto, CA, USA) to quantify the dose delivered through the acquisition of portal images. The dose distributions were calculated using the actual portal imaging parameters including gantry angle, collimator angle, couch angle, energy, and the total number of monitor units. The first part of the double exposure image (i.e., the treatment field image) was represented using the actual treatment field size and shape. The completed irradiated aperature outline (CIAO) was used for IMRT fields. The second part of the double exposure image was represented by the median collimator setting, since the open field size was set at the discretion of the therapist and was not consistent.

## III. RESULTS

Table 1 lists the mean and range of portal images (PI) and portal imaging monitor units (PI MU) per patient and per fraction for different (brainstem, nonbrainstem, CSI, and other) treatment sites. Only craniofacial (not spinal axis) portal images are included for CSI. The mean number of portal images acquired per patient was 58.8, an accumulation of orthogonal setup images, treatment field images, and open field images as part of the double exposure technique used at our institution. The mean number of portal images acquired per fraction was 1.9. The mean number of monitor units delivered through portal image acquisition was 173.3, with a mean of 5.6 monitor units per fraction.

**Table 1:** Mean and range of number of portal images (PI) and imaging monitor units (MU).

*Treatment Site*	*PI/Patient*	*PI/Fraction*	*PI MU/ Patient*	*PI MU/ Fraction*
Brainstem	50.3 (30–91)	1.6 (1.0–2.8)	149 (90–273)	4.8 (3.0–8.3)
Nonbrainstem	65.0 (56–69)	2.0 (1.7–2.1)	193 (163–207)	5.9 (4.9–6.3)
CSI^a^	81.3 (51–114)	2.7 (1.7–3.8)	236 (150–290)	7.7 (4.8–9.7)
Other	36.0 (31–45)	1.4 (1.1–1.8)	107 (93–135)	4.2 (3.3–5.4)
All	58.8	1.9	173.3	5.6

a Only craniofacial (not spinal axis) portal images are included.

Table 2 shows the mean and range of maximum, minimum, and mean portal imaging dose to the PTV, expressed as percentage of the prescribed dose, for the four different treatment sites. Because PTV was not defined for cranial step brain fields of CSI, the average imaging dose (3.8%) to the midline reference prescription point is presented. The maximum, minimum, and mean imaging doses to the PTV were 2.6%, 2.0%, and 2.3% for brainstem patients; 3.3%, 2.5%, and 2.9% for nonbrainstem patients; and 2.6%, 2.0%, and 2.4% for other patients.

**Table 2:** Portal imaging dose to the PTV (expressed as percentage of the prescribed dose).

*Treatment Site*	*PTV (maximum)*	*PTV (minimum)*	*PTV (mean)*
Brainstem	2.6 (1.2–4.3)	2.0 (0.9–3.6)	2.3 (1.1–3.9)
Nonbrainstem	3.3 (3.0–3.6)	2.5 (2.1–2.6)	2.9 (2.5–3.1)
CSI		3.8^a^ (2.5–5.0)	
Other	2.6 (2.2–3.4)	2.0 (1.3–2.7)	2.4 (1.8–3.2)
All	2.9	2.2	2.5

a PTV was not defined for cranial step brain fields for CSI, dose to the midline reference point is presented.

Table 3 demonstrates the mean and range of mean dose, expressed as percentage of the prescribed dose, to OARs due to portal imaging for the four different treatment sites. The average mean dose to the OARs through portal imaging was: brainstem 2.8%, optic nerves/ chiasm 2.6%, cochlea 2.6%, hypothalamus/pituitary 2.4%, temporal lobes 2.3%, thyroid 1.6%, and eyes 2.6%.

**Table 3:** Mean and range of mean dose (expressed as percentage of the prescribed dose) to OARs.

*OAR*							
*Tx Site*	*Brainstem*	*Optic Nerve/Chiasm*	*Cochlea*	*Hypothalamus/Pituitary*	*Temporal Lobes*	*Thyroid*	*Eyes*
Brainstem		2.3 (1.0–3.3)	2.3 (1.0–3.8)	2.3 (1.1–3.3)	2.2 (1.0–3.4)	1.2 (0.04–2.3)	2.6 (1.0–3.4)
Nonbrainstem	2.7 (2.3–2.9)	2.7 (2.3–3.0)	2.6 (2.3–2.9)	2.6 (2.3–3.0)	2.5 (2.1–2.8)	1.7 (0.1–2.7)	2.8 (2.4–3.1)
CSI	3.9 (2.4–5.5)	3.6 (2.0–5.1)	3.7 (2.2–4.9)			1.8 (1.4–2.5)	3.2 (1.8–5.2)
Other	1.6 (1.2–2.3)	1.9 (1.3–2.8)	1.7 (1.2–2.5)	2.1 (1.3–2.8)	1.8 (1.3–2.2)	2.0 (1.3–2.6)	1.9 (1.5–2.6)
All	2.8	2.6	2.6	2.4	2.3	1.6	2.6

Table 4 demonstrates the mean and range of mean dose, expressed as cGy, to OARs due to portal imaging for the four different treatment sites. The average mean dose to the OARs through portal imaging was: brainstem 153 cGy, optic nerves/chiasm 149 cGy, cochlea 144 cGy, hypothalamus/pituitary 132 cGy, temporal lobes 124 cGy, thyroid 85 cGy, and eyes 146 cGy.

**Table 4:** Mean and range of mean dose (expressed as cGy) to OARs.

*OAR*							
*Tx Site*	*Brainstem*	*Optic Nerve/Chiasm*	*Cochlea*	*Hypothalamus/Pituitary*	*Temporal Lobes*	*Thyroid*	*Eyes*
Brainstem		131 (53–196)	129 (56–227)	127 (59–193)	125 (55–204)	55 (2–140)	130 (56–203)
Nonbrainstem	157 (134–170)	164 (138–180)	155 (135–171)	158 (136–176)	142 (119–168)	102 (8–162)	167 (141–186)
CSI	206 (133–259)	201 (111–277)	204 (124–264)			110 (78–137)	186 (101–282)
Other	76 (55–105)	89 (58–128)	80 (56–112)	91 (58–124)	84 (67–100)	87 (59–115)	89 (68–117)
All	153	149	144	132	124	85	146

**Figure 1:**
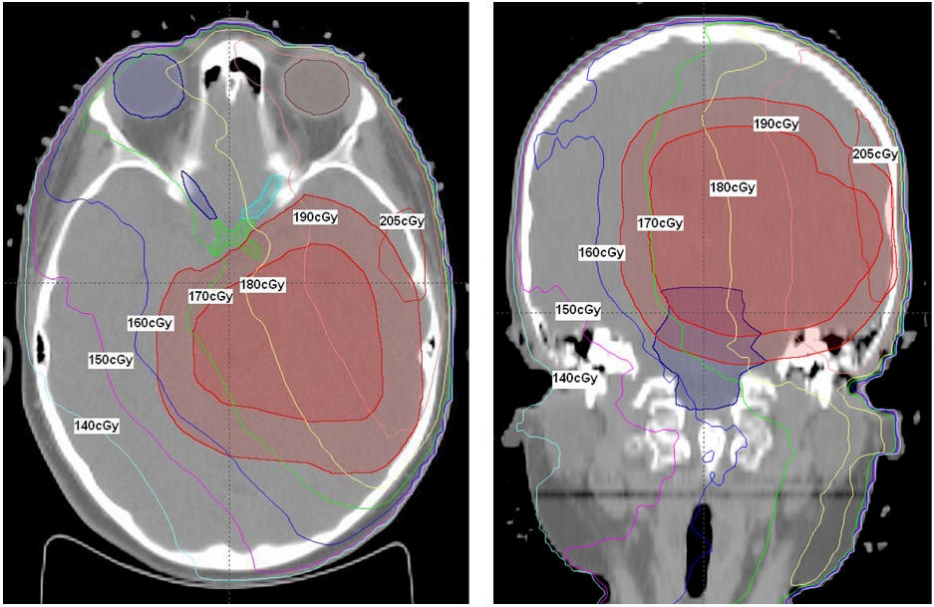
Illustration of the cumulative dose distribution from portal imaging for a nonbrainstem patient.

**Figure 2:**
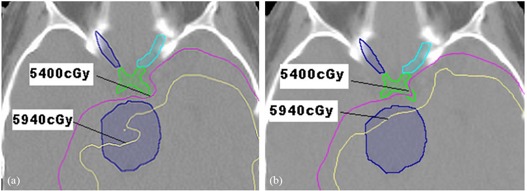
Dose to the chiasm and brainstem structures for a nonbrainstem patient: (a) treatment dose; (b) treatment plus portal imaging dose.

**Figure 3:**
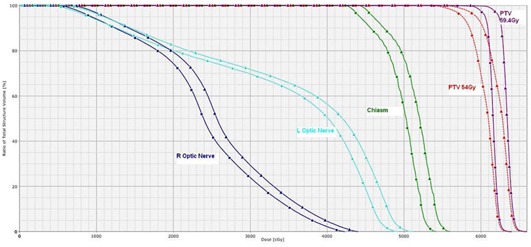
Comparison DVH curves for treatment dose (squares) versus treatment plus PI dose (triangles).

Treatment dose and treatment plus PI dose for a single nonbrainstem case is shown in Figs. 1, 2, and 3. Figure 1 illustrates the dose distribution due to portal imaging only. Figure 2 is a comparison of the treatment dose and the treatment plus portal imaging dose, Fig. 2 (a) and Fig. 2 (b), respectively. The increase in the dose to the chiasm and brainstem is apparent in Fig. 2 (b). Figure 3 shows comparison DVH curves for treatment dose (represented by squares) versus treatment plus PI dose (represented by triangles) for PTVs and OARs, including optic nerves and chiasm.

## IV. DISCUSSION

The aim of this study was to determine radiation dose to the PTV and OARs as a result of portal imaging of the craniofacial region in pediatric brain tumor patients treated with radiation therapy. It is observed from Table 1 that each patient received on average 58.8 portal images (173.3 MU) during the course of brain radiotherapy. The radiation dose from portal imaging is on average 2%–3% of the total prescribed dose for both PTV (Table 2) and surrounding OARs (Tables 3 and 4), indicating that an additional ~0.5–1 fraction equivalent of radiation treatment was delivered to those patients without being considered during treatment planning or documented in the dose distributions or dose‐volume histograms.

The extra 2%–3% of the total prescription dose might be acceptable for PTV coverage given the 5% dose uncertainty in the radiotherapy treatment process.^(^ ^8^ ^,^ ^9^ ^)^ However, the same amount of extra dose from portal imaging could easily push the dose to the surrounding OARs over the tolerance limit since, for many cases, the surrounding OARs were located next to PTV and already received close to the tolerance dose from the treatment beams (Figs. 2 and 3).

Portal imaging dose can be reduced by decreasing imaging frequency and lowering the number of monitor units for each exposure. Because imaging frequency is related to the quality of treatment,^(^ ^9^ ^)^ imaging frequency can be decreased only if the same confidence level of setup accuracy can be maintained. For example, in adaptive radiotherapy,^(^ ^10^ ^,^ ^11^ ^,^ ^12^ ^,^ ^13^ ^)^ portal imaging is performed more frequently in the first few fractions of the treatment to identify the random and systematic uncertainty. Once the systematic error is corrected, imaging frequency can be reduced so that the same treatment margin is still sufficient for the random errors.

Portal imaging dose to the OARs can be further reduced by decreasing the field size of both the open exposure and orthogonal setup fields, and by using single exposure whenever feasible. However, because the accuracy of patient setup using portal imaging depends on the available anatomic structures in imaging field of view, overcropping the field size for portal imaging might significantly compromise the accuracy of the evaluation of the patient setup.

Further dose reduction may be achieved by implementation of a portal imaging policy which addresses image acceptance criteria, detailing isocenter shift guidelines. Since our institution had not implemented such a policy, shifts of 1–2 mm were made to align the isocenter of the orthogonal portal images with the DRR isocenter. Additional portal images were acquired after such shifts were made. Implementation of a policy requiring shifts be made only if alignment is off by > 2 mm would eliminate re‐imaging and reduce dose from portal imaging.

Portal imaging dose would be reduced by imaging each treatment field without the patient in the room. In as much as our institution currently participates in several national protocols which require a portal image of each treatment field, our portal images are acquired with the patient in the beam.

Gantry‐mounted kV imaging systems have been available for the past few years as an image‐guided radiation therapy (IGRT) tool, utilizing kV planar imaging, as well as kV CBCT.^(^ ^14^ ^,^ ^15^ ^)^ Portal imaging dose to the patient is decreased with kV planar imaging versus MV imaging, and kV imaging provides better contrast for patient setup verification. The kV CBCT has the advantage over planar imaging in that kV CBCT provides detailed three‐dimensional volumetric information useful for patient setup verification. The dose resulting from kV CBCT is comparable to dose from conventional multiple MV setup fields.^(^ ^16^ ^)^

While the most obvious benefit of reduction in PI dose is likely to happen in pediatric brain tumor patients, the same general principles hold good for adult brain tumor patients, too. However, the extent of benefit from reduction in PI is likely to be less, based on mathematical modeling.^(^ ^17^ ^)^ The biggest advantage in adults would likely be in large tumors located in anterior temporal lobe where there is a risk of radiation injury to multiple OAR including optic nerve, chiasm, retina, brainstem, pituitary gland, and cochlea. Precise documentation of the dose and long‐term monitoring of the adult brain tumors would be needed to show the extent of benefit from reduction in PI measures.

## V. CONCLUSIONS

The dose from PI while treating pediatric brain tumors is significant. This additional dose may result in exceeding the tolerance limit of many critical structures, and has the potential to increase the risk of late complications and secondary malignancies. Dose contributions from PI should be considered in the final documented dose. Based on these results, we have implemented changes in PI practices at our institution to lower the imaging dose and volume, when feasible, and will update the results in future.

## References

[1] Mueller S and Chang S . Pediatric brain tumors: current treatment strategies and future therapeutic approaches. Neurotherapeutics 2009;6(3):570–86. 19560746 10.1016/j.nurt.2009.04.006 PMC5084192

[2] Twombly R . Pediatric brain tumor survivors, physicians, and researchers face long‐term challenges. J Natl Cancer Inst. 2009;101(13):908–10. 19549957 10.1093/jnci/djp190

[3] Skowronska‐Gardas A . A literature review of the recent radiotherapy clinical trials in pediatric brain tumors. Rev Recent Clin Trials 2009;4(1):42–55. 19149762 10.2174/157488709787047567

[4] Miralbell R . Lomax A, Cella L, Schneider U. Potential reduction in the incidence of radiation‐induced second cancers by using proton beams in the treatment of pediatric tumors. Int J Radiat Oncol Biol Phys. 2002;54(3):8234–8239. 10.1016/s0360-3016(02)02982-6 12377335

[5] van Kranen H , Westerman A , Berq R , et al. Dose‐dependent effects of UVB‐induced skin carcinogenesis in hairless p53 knockout mice. Mutat Res. 2005;571(1–2):81–90. 15748640 10.1016/j.mrfmmm.2004.07.018

[6] Shuryak I , Sachs RK , Brenner DJ . A new view of radiation‐induced cancer. Radiat Prot Dosimetry 2011;143(2–4):358–64. 21113062 10.1093/rpd/ncq389 PMC3108273

[7] Narayana A. , Yamada J , Berry S , et al. Intensity‐modulated radiotherapy in high‐grade gliomas: clinical and dosimetric results. Int J Radiat Oncol Biol Phys. 2006;64(3):892–97. 16458777 10.1016/j.ijrobp.2005.05.067

[8] International Commission for Radiation Units and Measurement . Determination of absorbed dose in a patient irradiated by beams of X or gamma rays in radiotherapy procedures. ICRU Report 24. Bethesda (MD): ICRU; 1976.

[9] Kutcher G J , Coia, L , Gillin M , et al. Comprehensive QA for radiation oncology: report of AAPM Radiation Therapy Committee Task Group 40. Med Phys. 1994;21(4):581–618. 8058027 10.1118/1.597316

[10] Bel A , van Herk M , Bartelink H , Lebesque JV . A verification procedure to improve patient set‐up accuracy using portal images. Radiother Oncol. 1993;292:253–60. 10.1016/0167-8140(93)90255-7 8310153

[11] Yan D , Wong J , Vicini F , et al. Adaptive modification of treatment planning to minimize the deleterious effects of treatment setup errors. Int J Radiat Oncol Biol Phys. 1997;38(1):197–206. 9212024 10.1016/s0360-3016(97)00229-0

[12] Yan D , Ziaja E , Jaffray D , et al. The use of adaptive radiation therapy to reduce setup error: a prospective clinical study. Int J Radiat Oncol Biol Phys. 1998;41(3):715–20. 9635724 10.1016/s0360-3016(97)00567-1

[13] Herman MG . Clinical use of electronic portal imaging. Sem Radiat Oncol. 2005;15(3):157–67. 10.1016/j.semradonc.2005.01.002 15983941

[14] Chang J , Yenice KM , Narayana A , Gutin PH . Accuracy and feasibility of cone‐beam computed tomography for stereotactic radiosurgery setup. Med Phys. 2007;34(6):2077–84. 17654911 10.1118/1.2731031

[15] Murphy M , Balter JM , Balter S , et al. The management of imaging dose during image‐guided radiotherapy: Report of the AAPM Task Group 75. Med Phys. 2007:34(10):4041–63. 17985650 10.1118/1.2775667

[16] Ding GX and Coffey CW . Radiation dose from kilovoltage cone beam computed tomography in an image‐guided radiotherapy procedure. Int J Radiat Oncol Biol Phys. 2009;73(2):610–17. 19147025 10.1016/j.ijrobp.2008.10.006

[17] Hall EJ . The impact of protons on the incidence of second malignancies in radiotherapy. Technol Cancer Res Treat. 2007;6(4 Suppl):31–34. 10.1177/15330346070060S405 17668949

